# Quantifying isocenter measurements to establish clinically meaningful thresholds

**DOI:** 10.1120/jacmp.v16i2.5183

**Published:** 2015-03-08

**Authors:** Travis R. Denton, Lisa B.E. Shields, Jonathan N. Howe, Aaron C. Spalding

**Affiliations:** ^1^ The Norton Cancer Institute Radiation Center Louisville KY; ^2^ Associates in Medical Physics, LLC Greenbelt MD; ^3^ Norton Neuroscience Institute Louisville KY; ^4^ The Brain Tumor Center, Norton Healthcare Louisville KY USA

**Keywords:** stereotactic, radiosurgery, isocenter, linear accelerator, quality assurance

## Abstract

A dataset range of isocenter congruency verification tests have been examined from a statistical perspective for the purpose of establishing tolerance levels that are meaningful, based on the fundamental limitation of linear accelerator isocentricity and the demands of a high‐precision stereotactic radiosurgery program. Using a laser‐defined isocenter, a total of 149 individual isocenter congruency tests were examined with recorded values for ideal spatial corrections to the isocenter test tool. These spatial corrections were determined from radiation exposures recorded on an electronic portal imaging device (EPID) at various gantry, collimator, and treatment couch combinations. The limitations of establishing an ideal isocenter were quantified from each variable which contributed to uncertainty in isocenter definition. Individual contributors to uncertainty, specifically, daily positioning setup errors, gantry sag, multileaf collimator (MLC) offset, and couch walkout, were isolated from isocenter congruency measurements to determine a clinically meaningful isocenter measurement. Variations in positioning of the test tool constituted, on average, 0.38 mm magnitude of correction. Gantry sag and MLC offset contributed 0.4 and 0.16 mm, respectively. Couch walkout had an average degrading effect to isocenter of 0.72 mm. Considering the magnitude of uncertainty contributed by each uncertainty variable and the nature of their combination, an appropriate schedule action and immediate action level were determined for use in analyzing daily isocenter congruency test results in a stereotactic radiosurgery (SRS) program. The recommendations of this study for this linear accelerator include a schedule action level of 1.25 mm and an immediate action level of 1.50 mm, requiring prompt correction response from clinical medical physicists before SRS or stereotactic body radiosurgery (SBRT) is administered. These absolute values were derived from considering relative data from a specific linear accelerator and, therefore, represent a means by which a numerical quantity can be used as a test threshold with relative specificity to a particular linear accelerator.

PACS number: 87.53Ly, 29.20.Ej, 87.56.Fc

## I. INTRODUCTION

Stereotactic radiosurgery (SRS), by definition, is the delivery of a large therapeutic dose of radiation to an accurately localized target.^1^, ^2^, ^3^, ^4^, ^5^, ^6^, ^7^, ^8^ This results in an ablative dose of radiation at the target with a steep dose gradient to minimize damage to tissues outside of the target.^9^, ^10^, ^11^, ^12^ SRS treatments are generally associated with small intracranial targets, and recently the term stereotactic body radiosurgery (SBRT) was developed to describe stereotactic‐guided radiotherapy to extracranial sites.^13^, ^14^, ^15^, ^16^, ^17^

The linear accelerator's isocenter is the common point shared between the mechanical axes of the gantry, treatment couch, and collimator with both the treatment beam axis and the imaging isocenter. SRS requires precise alignment of these axes such that the isocenter remains constant while any of these components change position. In 1987 Winston and Lutz described an isocentricity test to establish congruency of these axes.^18^, ^19^, ^20^ The test involved determining the coincidence of the treatment isocenters of the linear accelerator and associated imaging system. A small phantom containing an internal radioopaque spherical marker with a diameter of 5 mm is aligned via external crosshairs using an in‐room laser localization system. Radiation exposures are made at various gantry, collimator, and treatment couch combinations, and the offset may be ascertained by measuring the center of the radiation field in relation to the center of the spherical marker.

The Winston‐Lutz (WL) test was originally developed to provide QA for a single fraction of cranial SRS using a rigid invasive headframe attached by a neurosurgeon with the patient under local anesthesia. The localization of the target with this technique relies on aligning the lasers to the frame with the assumption that, when treating the patient, the frame has not shifted from simulation due to the pins screwed into the skull. These headframes produce imaging artifacts, and therefore imaging verification of the patient position was not possible. Now radiolucent cranial and body immobilization devices allow photon imaging to verify proper patient position. Therefore, this test has been modified from the use of film with the advent of on‐board imagers as the image acquisition device.^21^, ^22^, ^23^, ^24^

Image‐guidance in radiotherapy added tertiary considerations to define a congruent isocenter with the possible inclusions of on‐board megavoltage (MV) and kilovoltage (kV) imaging devices and other ancillary imaging platforms, such as ExacTrac.^25^, ^26^, ^27^ Furthermore, traditional Winston‐Lutz isocenter alignment tests have largely been qualitative in nature, and the relative information derived from visual inspection of the images. This presents a challenge in analyzing trend‐based behaviors due to a lack of quantitative information recorded.

The present study attempted to formalize the inclusion of imaging components to the Winston‐Lutz test with a statistically driven examination of data points to derive meaningful action limits to the routine performance of this quality assurance test. For the course of just over one calendar year, a total of 149 test results were analyzed, including MLC and cone‐based isocenter alignment examinations.^28^, ^29^, ^30^, ^31^ This allowed for the quantification of some of the variables affecting the precision of the isocenter alignment, specifically, gantry sag, MLC offset, couch walkout, and daily setup uncertainties.

Thresholds have been suggested to be established as being within specification of a baseline value.^(32)^ This study describes both a mechanism by which machine‐specific thresholds may be derived, and establishes absolute values for threshold definitions based on data relative to itself through a statistically driven procedure. We obtained absolute values to which action limits may be applied based on machine‐specific relative baseline data, instead of considering only the relative baseline data with arbitrary action limits.

## II. MATERIALS AND METHODS

### A. Data collection

The Winston‐Lutz test was conducted on a total of 149 measurements obtained over 370 days. Of these datasets, 100 were complete tests which were performed for SRS or SBRT and utilized the treatment table, collimator, and gantry angles (Table 1). Each entry reflects a series of images acquired during a full isocenter congruency test. Each dataset consisted of a full or subset of these data points. Fields 13 and 14 (not listed in the table) complete the dataset for each test, and correspond to gantry and table angles of zero degrees with collimator rotated to 90° and 270°, respectively. A total of 38 datasets consisted of abbreviated measurements, specifically, a test performed without the variation of couch angles. Eleven datasets underwent tests with equivalent field definitions and used a conical collimator instead of field sizes defined by high‐definition MLCs.

**Table 1:** Definition of field labeling for isocenter congruency tests

*Definition of Fields*					
*Gantry Angle (°)*			*Table Angle (°)*		
	0	45	90	270	315
0	Field 1	Field 2	Field 3	Field 4	Field 5
90	Field 6				
180	Field 7	Field 8	Field 9	Field 10	Field 11
270	Field 12				

### B. Equipment

The linear accelerator used in this study was a Novalis Tx (Varian Medical Systems, Palo Alto, CA) and BrainLAB AG (Feldkirchen, Germany). This included BrainLAB's ExacTrac system and Varian's On‐Board Imaging (OBI).^33^, ^34^, ^35^ The linear accelerator was equipped with high‐definition MLC (HD120 MLC) suited for SRS targets as small as 5 mm. Radiation exposures were recorded on a Varian high‐definition amorphous silicon‐based EPID panel (Portal Vision, aS1000, Varian Medical Systems).

The alignment phantom was a Winston‐Lutz pointer phantom (BrainLAB) which consisted of crosshairs for laser‐guided positioning with an embedded radiopaque BB (5 mm diameter) at the crosshair‐defined center of the phantom. For MLC‐defined and cone‐based MV Winston‐Lutz images, an exposure was made utilizing a square field defined either by the collimation from a $1.5\times 1.5\;{\text{cm}}^{2}$ field size defined by MLCs or a circular field defined by conical collimators at a variety of gantry, couch, and collimator angles with the alignment phantom positioned such that the radiopaque BB would fall within the radiation field with proper alignment. An analysis of each image was performed in DoseLab Pro (Mobius Medical Systems, LP, Tampa, FL) wherein the radiation field edge threshold was determined along with the edge of the center object (i.e., the radiopaque BB). The centroid of each of these two objects was then determined using a proprietary algorithm reporting accuracy to within a third of a pixel.^(36)^ An offset calculation was then performed between the two centroids.

For MLC‐defined spoke shot films, electronic portal imaging device (EPID) images were taken as a series of thin slit fields formed by bringing both banks of MLCs close to each other. Exposures were then made at various collimator angles forming a star pattern. A cumulative image was combined from these fields and analyzed using DoseLab Pro. The gantry position was constant, and there was no phantom intermediate between the gantry head and imaging panel. This analysis was performed by sketching a ray line through the measured center of each slit (i.e., each exposure). The smallest circle that intersected each ray at least once was taken as the quality metric for this test. Numerical assignments for baseline measurements in periodic quality assurance were derived by noting the diameter of this circle.

Varian's IsoCal geometric calibration system was applied to images acquired with the MV imagers to compensate for mechanical defections and arm position errors that occurred for the linear accelerator as a function of gantry angle.^(37)^ Independent testing was performed to evaluate the accuracy of the IsoCal calibration data at a sampling of gantry angles consisting of the four cardinal gantry angles with a comparison of two images for each gantry angle — one acquired of the alignment phantom setup with the MV panel, and another obtained from an exposure to radiochromic film secured on a fixed surface independent of the gantry and panel. This comparison of the digital‐ and film‐based images analyzed for congruency of the radiation field and BB‐defined isocenters showed, on average, a difference of 0.10 mm and maximum and minimum differences of 0.18 mm and 0.01 mm, respectively, which is comparable to values reported in studies formally evaluating the accuracy of IsoCal.^38^, ^39^, ^40^

The Winston‐Lutz test was performed at a frequency and/or preceding radiotherapy treatments prescribed by the protocols established by the treatment team and specific to the nature of the treatments being performed. In addition, daily testing consisted of brief, simplified alignment tests. The patient alignment tools tested depended on the nature of the imaging appropriate for those treatments and included testing of the congruency of the radiation isocenter, laser‐guided positioning system, MV and kV imaging devices, and ExacTrac imaging components by taking simultaneous images of the WL phantom at predetermined gantry positions. In both tests and patient alignment situations, precursor alignment was performed using the in‐room lasers with image‐guidance following. Congruency was verified during testing between all of the appropriate imaging platforms with action levels defined as 0.7 mm for ExacTrac with EPID and 1 mm for kV imaging with EPID. We did not observe misalignment beyond either of these action levels in any of the 149 WL tests. Therefore, we focused on the analysis of EPID‐based images, as these are used to define the laser and WL alignment phantom. We plan to proceed with the analysis of kV and ExacTrac congruency with EPID in future investigations to determine if these action levels are appropriate.

### C. Statistics

A two‐sample assuming unequal variances *t*‐test was performed in this study, with a defined significance level of 0.05.

## III. RESULTS

### A. Daily positioning errors and global uncertainty value

To determine daily positioning setup error of the isocenter test tool from measurements, each data point was examined as to whether the pointer positioning could have been improved from the imaging results. Using images with complementary information, each spatial dimension was evaluated to determine whether or not the alignment of the test tool could have been improved, with the requirement that all images containing information for the dimension under analysis confirm the improvement. If the images provided an affirmative to this, a daily setup error spatial correction was deduced using the minimum spatial correction for that test as determined from all images contributing information for that dimension. This three‐dimensional spatial correction for each dimension and dataset was then applied to the data to determine the positioning error to establish a so‐called corrected data collection determined from an uncorrected one. The average spatial corrections with the daily setup bias removed from all of the datasets are presented in Table 2. Following the isolation of the datasets from daily positioning error bias, an evaluation of the statistics from all datasets yielded an average, median, standard deviation, and mode of 0.58, 0.57, 0.21, and 0.54 mm, respectively.

**Table 2:** Magnitude of corrections needed to remove day‐to‐day test tool setup variation

*Average Spatial Correction (mm)*	
*Direction*	*Average*
Left–Right	−0.06
Superior–Inferior	−0.13
Anterior–Posterior	0.19
Magnitude of Correction	0.38

**Figure 1:**
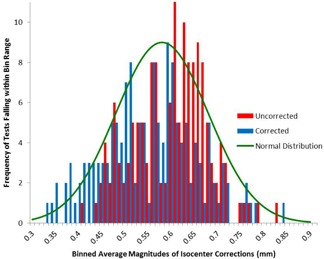
Histogram with and without setup error. The blue curve is a representation of binning test results of daily positioning setup unbiased data, while the red curve is a histogram of the data without the correction applied to remove daily positioning error.

The sum of the datasets was compiled in a histogram displaying both the results with and without the daily positioning setup error (Fig. 1). Following the removal of the daily setup positioning error, the average and 1 SD of the total magnitude of shift required to align the laser‐defined isocenter to the radiation‐defined isocenter was 0.58±0.21 mm. Figure 1 shows a very close approximation in behavior and distribution to a Gaussian distribution. A total of 71.8% of the data points fell within 1 SD (0.21 mm), and 98.7% of the data points fell within 2 SDs (0.42 mm). There was a significant difference between the magnitudes of shifts needed between the two curves, with

(1) $$P{\left(T\leq t\right)}_{\text{two}‐\text{tail}}5.52\times 10^{-6}(p<0.05)$$

The other component of this investigation allowed for small positioning deviations in the daily positioning of the test tool. After reviewing the device positioning correction per test, the average magnitude with 1 SD of three‐dimensional shift was 0.38±0.25 mm.

**Figure 2:**
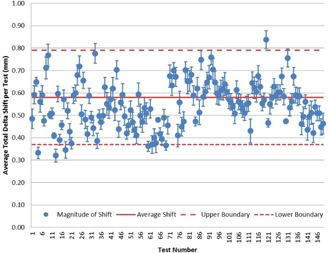
Data points with error bars of 2 SDs. Average total magnitude of shift required to correct for the incongruency of isocenter of the phantom center and radiation field for each test with the daily setup error removed from the data. Each data point is displayed with standard error calculated from each of the 14 measurements used to calculate the mean. Additionally, for the dataset as a whole, the mean±1 SD is displayed. Working under the assumption that the histograms approximate Gaussian behavior, it can be approximated that the error bars should encompass 95% of the data points.

Figure 2 displays the average total magnitude of correction shifts (average=0.58 mm) needed for each dataset. The upper and lower boundaries are defined by 2 SDs (average=0.42 mm) of the setup error for the daily positioning error unbiased data (i.e., data corrected for the daily positioning error).

### B. Gantry sag

**Figure 3:**
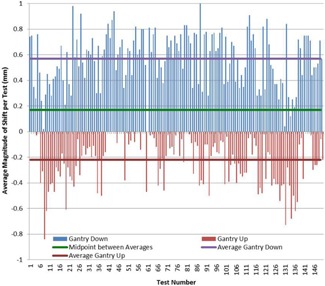
Gantry sag. Total magnitude of corrected shift with the gantry angle above and below the test device (i.e., with the gantry in up and down positions) in a one‐dimensional representation. In an attempt to quantify gantry sag, the midpoint value between these extremes was 0.17 mm shifted toward the gantry 180° position. The difference from this midpoint to either extreme was 0.4 mm, leading to the indication that the gantry sag magnitude may be quantified to 0.4 mm.

Figure 3 illustrates the impact of gantry sag on the longitudinal shift requirements noted by comparing the gantry 0° (IEC scale) (gantry up) and 180° (gantry down) measured longitudinal shift requirements for isocentricity between the laser and radiation‐defined isocenters. The daily positioning setup uncertainty was removed from the data points to isolate the gantry sag variable given the available data.

The average gantry 180° shift was 0.57 mm, while the average gantry 0° shift was −0.22 mm. The midpoint of these two values was 0.17±0.4 mm. The standard deviation of the gantry down data for the 149 datasets was 0.19 mm, while the standard deviation for the gantry up was 0.21 mm.

**Figure 4:**
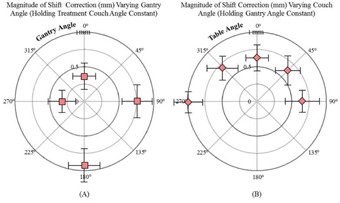
Variable couch and gantry variable combined gantry constant. (a) Effect of gantry sag on radiation‐defined vs. laser‐defined isocenters as shown by radially plotting the vector of magnitude shift correction (mm) while varying gantry angle $\left(\text{couch angle}=0^{\circ }\right)$. The circular perimeter was 1 mm. (b) Average magnitude of correction shifts needed for all datasets for various couch angle positions holding all other machine parameters constant plotted on a radial plot with a 1 mm outer diameter.

Figure 4(a) represents the magnitude of correctional shift needed for fields with variable gantry and static table positions.

### C. MLC offset and couch walkout

Two methods of measuring MLC walkout were employed in this study. For the first test, eight exposures were made with only a thin slit (5 mm) of radiation revealed by the MLCs. A line extended through each slit, and a circle was generated. The quantification of this test was in the generation of this circle which had a diameter that was able to touch all of the lines from each spoke for at least one intersection point. The magnitude was measured as 0.16 mm.

The second method consisted of investigating each MLC field defined dataset (n=138) with gantry and couch positions held at values of 0° while image acquisitions were made at collimator angle of 0°, 90°, and 270°. The gantry was in an up position, and the couch was in line with the gantry stand. Holding all parameters constant, with the exception of the collimator angle for MLC‐defined fields, the difference may be attributed to the combination of both MLC offset and walkout. Table 3 summarizes the data compiled from daily measurements.

For quantification of the MLC offset, the collimator is considered at three positions (0°, 90°, and 270°). The degree of offset and walkout is measured relative to the collimator at 0° by noting the magnitude of correction needed at either extreme (90° and 270°). The magnitude of correction for the 0° collimator position was noted to fall between the magnitudes of correction for 90° and 270°, and the difference between the magnitudes for the extremes was the quantified measure of the effect of collimator offset and walkout.

An illustration of the effect of couch walkout is shown (Fig. 4(b)). The magnitude of corrections is demonstrated while varying couch positions and holding other machine parameters constant. This data ranged from a magnitude of correction needed of 1.08 mm on average for the 149 datasets for couch angle of 270° to a value of 0.36 mm for a couch angle of 0°. The couch was measured with the lowest correction needed at the couch angle of 0°, while it was the worst at couch extremes of 270° and 315° (1.08 mm and 0.49 mm).

**Table 3:** MLC offset and collimator walkout determination

*Measured Corrections Needed with Variable MLC‐defined Fields*			
	*Total Magnitude (mm) G0T0*	*Total Magnitude (mm) G0T0C90*	*Total Magnitude (mm) G0T0C270*
Average	0.38	0.3	0.4
Difference with G0T0		−0.08	0.02
Average Difference			−0.03
Magnitude of Difference			0.1

### D. Action limits

**Figure 5:**
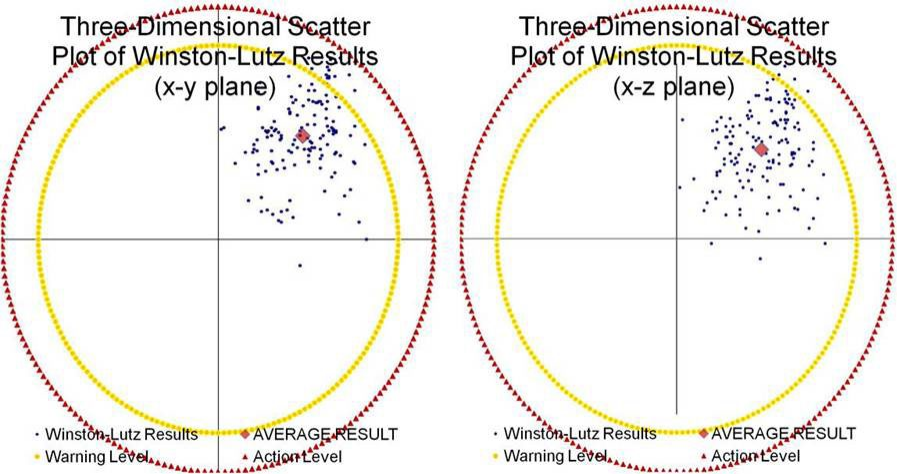
3D plot. Two‐dimensions shown of a three‐dimensional scatter plot. The blue data points represent the isocenter as defined by the lasers during the test tool alignment for each of the considered 149 datasets. The vector directions were defined as positive x=lateral left, positive y=towards gantry stand, and positive z=down. The origin of this plot was the actual radiation isocenter. The red data point represents the average measured value of magnitude of noncongruency between the isocenters seen from the center of the test tool to the center of the radiation field. The outer circular perimeters represent a 1.25 mm warning level and a 1.50 mm action level.

A global evaluation of each of the 149 datasets is presented in a three‐dimensional illustration where the x, y, and z positions are the spatial corrections needed to align the radiation defined isocenter from the laser defined isocenter (Fig. 5). The parameters for both the schedule action level and the immediate action level were evaluated. An average result was compiled from each of these datasets which demonstrated an offset of the congruency of the isocenters. Using the concept of standard deviations for two separate action levels, we defined a schedule action level as

(2) 0.58(±0.42+0.25)mm=1.25 mm

and an immediate action level of

(3) 0.58(±0.42+0.50)mm=1.50 mm

This first term (0.58 mm) was the average magnitude of correction needed for the datasets considered after the exclusion of the effects of day‐to‐day setup variation. The second term (±0.42 mm) was two standard deviations of the data after the exclusion of day‐to‐day setup variation which was found to encompass all of the effects of gantry sag, MLC offset, collimator walkout, and couch walkout. The final term (±0.25 or ±0.50 mm) was either 1 or 2 SDs, which included the day‐to‐day setup variations of the test tool for either the schedule action level or the immediate action level, respectively.

## IV. DISCUSSION

In order to fully appreciate numerical results from isocenter analyses, it is important to understand and isolate the limitations of isocenter congruency based on independent confounding variables, including daily positioning errors of the test tool, gantry sag, couch walkout, and MLC offset. These effects are alike in that they all add to the uncertainty of measurements, but are not correctable beyond a fundamental limitation determined by mechanical factors of the linear accelerator's construction. This study attempted to quantify the magnitude of these effects separately and then evaluate their additive effect on measurement uncertainty. After the magnitude of the uncorrectable effects was accounted for, a global threshold was established based on clinical data which determined action limits based on numerical results measured during a routine test.

The datasets that have been examined in this study are differentiated based on the type of treatment employed. Abbreviated tests are performed for SBRT that do not include any couch positions other than the home position, specifically, with the couch longitudinally in line with the linear accelerator's gantry stand. Furthermore, a cone‐based measurement set would not need to include the variation of collimator angles because of the inherent symmetry of cone‐based treatments.

During normal day‐to‐day measurement of isocentricity, there exists a bias due to test tool setup error (i.e., daily positioning errors). In this study, we attempted to isolate this error. The error was determined by noting image sets that offered redundant information in each dataset and whether a spatial correction would have improved the results. The appropriate geometric direction and magnitude of the correction that could have been applied was established (anterior‐to‐posterior, left‐to‐right, or superior‐to‐inferior). It is important to note that each correction was determined for each dataset and removed to obtain daily setup unbiased data of all datasets. The corrections required to remove day‐to‐day setup variations offer an indication of the magnitude of the error that may be propagated from the variation in setup of the test device and, thus, can offer some insight to the scope of uncertainty that we can afford for our test's sensitivity.

Given the distribution of the data presented in Fig. 1, it is reasonable to deduce that standard deviations may provide an appropriate approach in establishing associated thresholds with measured error. Furthermore, based on the assumption that it very nearly approximates a normal distribution, 95% of the data points should be included within 2 SDs where 1 SD=0.21.

Gantry sag refers to the mechanical displacement of the radiation isocenter at different gantry rotational positions.^40^, ^41^, ^42^ Gantry sag has been observed for all isocentric linear accelerators, but remains an effect that is highly dependent on the particular linear accelerator of interest.^29^, ^41^, ^43^, ^44^ For this reason, measuring gantry sag for the specific linear accelerator in question was a relevant step in establishing isocentricity measurement thresholds. Based on the comparison to daily average values, the magnitude of gantry sag resulted in a mechanical shift of radiation isocenter of 0.4 mm as measured specifically for this linear accelerator.

Using complementary image acquisitions at variable gantry angles, the effect from gantry sag may be quantified for inclusion in consideration of the fundamental limitations of measuring and defining the congruency between radiation‐ and laser/image‐defined isocenters for a linear accelerator. This is not to say that the magnitude of observed gantry sag (0.4 mm) changes on the order of millimeters day‐to‐day. Rather, gantry sag is a fairly constant and stable effect. The argument above quantifies the gantry sag effect to submillimeter values (specifically, an average measurement of 0.4 mm). Some of the noise from this data is the result of some limitations in accurately removing all setup uncertainty in the isocenter pointer phantom. This assertion is made with the assumption that the effect of gantry sag is evenly distributed between the two considered gantry angles. In reality, gantry sag is not evenly distributed among gantry angles, weighing more heavily on the gantry 0° position. This is seen in Fig. 3 by comparing the gantry down data versus the gantry up data with an error gradient along the rotation from up to down.

If the 0.4 mm midpoint represents a global indicator of the gantry sag magnitude, then it may be concluded that the effect of gantry sag on radiation‐ versus laser‐defined isocenters is within the statistical daily deviation of the global variations. If the average magnitude of correction needed is 0.58 mm with the removal of daily positioning errors, then the magnitude of gantry sag is less than this value. Therefore, gantry sag has a magnitude of effect within the average measured correction. Since this gantry sag magnitude is less, it may be concluded that no corrections should be necessary to counter‐effect gantry sag for day‐to‐day measurements. Alignment of the lasers should be performed such that the average deviation between isocenters is minimized. In this respect, the longitudinal shift between the anterior‐to‐posterior and posterior‐to‐anterior fields should be opposite in vector direction, and the average between the two should approach zero.

This study investigated image acquisitions taken with variable MLC defined collimator angles to quantify the magnitude of the contributor to the fundamental limitation of isocenter congruency due to MLC offset and walkout. MLC offset is defined as the degree of misalignment of the MLCs with respect to the collimators.^45^, ^46^ The recommended protocol for analyzing MLC offset and walkout is to perform a “spoke” or “star” shot using MLCs to define a narrow slit of exposure at various collimator angles.^32^, ^47^ This test and the isocenter congruency test have been completed per the guidelines of the American Association of Physicists in Medicine (AAPM) professional task group recommendations.^(32)^ The recommended tolerance for this test allows for no larger than 1.0 mm of measured deviation. With the measured results using the MLC‐based spoke shot test measured at 0.16 mm and the isocenter congruency test measuring values of 0.1 mm for the MLC offset and walkout portion of the dataset (Table 3), these tests display results that are well within agreement and within recommended tolerance.^(32)^

The treatment couch for a linear accelerator reflects another isocentric component and has its own associated mechanical limitations of isocenter accuracy.^23^, ^30^ Since this is another fundamental limitation of the isocenter definitions, it is important to take these results into account when characterizing tolerance values for the test. The couch walkout results in a large degree of dealignment for the congruency of the linear accelerator and imaging isocenters. The measured magnitude is one that must be taken into account with the threshold values established for this test.

In an attempt to define a clinically meaningful tolerance based on quantifiable history and understanding of the fundamental limitations of the linear accelerator, it should be noted that the average total magnitude of shift required to align the laser‐defined isocenter to the radiation‐defined isocenter is 0.58 mm, following the removal of the daily setup positioning error. Figure 1 showed a very close approximation in behavior and distribution to a Gaussian‐type (normal) distribution. Thus, we can approximately assert that 95% of the datasets will fall within 2 SDs of this dataset. One SD was measured to be 0.21 mm. Thus, 95% of datasets are expected to fall within

(4) 0.58±(2×0.21)=0.58±0.42 mm

A maximally accepted value will then be 1.0 mm given the datasets with daily positioning error misalignment isolated from the data. (This value is compared to the Varian Customer Acceptance Procedure (CAP) isocenter test value for a Varian Novalis Tx linear accelerator of central axis within 0.5 mm coincidence from radiation to imaging isocenter for gantry and/or collimator rotations and 0.75 mm for couch rotations). This assertion agreed very well with our observations that 98.7% of the data points fell within 2 SDs, lending credibility to this use of standard deviations as a threshold defining tool.

The other component of this investigation allowed for small deviations in the daily positioning of the test tool. After reviewing the device positioning correction per test, the average magnitude of three‐dimensional shift was 0.38 mm. One SD of this data was 0.25 mm. Unlike the other variables that were considered, this effect was cumulative and must contribute to the test threshold in addition to the 1.0 mm. This may be performed using a first and second tier of 1 and 2 SDs (0.25 and 0.50 mm, respectively).

Both the scheduled action level (or warning level) and an immediate (or stop treatment) action level are incorporated into global threshold values. The schedule action level (the lower of the two thresholds) should exist as a “red fag” with the test operators. If this value is exceeded, treatment may proceed but with the understanding that the medical physics staff are notified of this fag. The data should be investigated by the medical physicists, with a correction to the laser‐defined isocenter if deemed appropriate. Exceeding this level requires scheduled maintenance or investigation, with the understanding that mitigation of the cause will take place. The value associated with the scheduled action level is 1.25 mm which considers gantry sag, MLC offset, collimator walkout, couch walkout, and one level of magnitude of day‐to‐day setup variation of the test phantom. The immediate action level functions as a stop treatment level. If it is exceeded, adjustments should be made immediately to reduce the isocenter congruency results to below threshold prior to further patient treatment. This value is 1.50 mm and includes gantry sag, MLC offset, collimator walkout, couch walkout, and a high level of consideration of day‐to‐day setup variation.

In addition to considering daily deviations, 1 SD of the positioning error data may be used to establish a warning level magnitude, while 2 SDs may be used to determine an action level magnitude: 0.58+0.42+0.25 mm and 0.58+0.42+0.50 mm, respectively. The result of establishing this threshold (as seen most clearly by the perimeter lines in Fig. 5) are tolerance values that blanket the datasets to a degree that allows neither too loose of a constraint on the test or too tight of one. Failure to meet this balance would result in a test that is either clinically meaningless with all points passing effortlessly without the benefit of identifying true errors for the linear accelerator's isocenter, or a test that is not clinically realistic or practical.

It is important to note the evolution of threshold values for isocentricity tests. These threshold values were first established in professional recommendations as absolute values applicable to linear accelerators.^18^, ^19^, ^47^ This was fundamentally limited, however, due to the great deal of differences between different linear accelerators in both design and application. For example, a machine assigned for conventional three‐dimensional conformal patient treatments does not have the same stringent requirement for congruency of the radiation isocenter and image guidance systems. One solution to this was to split the absolute thresholds to separate values for non‐SRS/SBRT and SRS/SBRT machines.^(48)^

This solution failed to take into account trends of specific machines which may be very valuable tools in assessing the “normal” functioning of a linear accelerator. It was for this reason that suggestions for definitions of threshold values were revised to compare with baseline data (i.e., relative comparison vs. absolute tolerances).^(32)^ By utilizing baseline values, outliers in data points from measurement to measurement become highly convenient indicators of abnormal behavior of a treatment system and, therefore, suggestive of possible errors that could negatively impact treatment.

If relative baselines are solely considered as threshold values, then the advantage of absolute tolerances are lost. When the baseline values are used as tolerance definitions, as long as the test results are always grossly incongruent, then they still meet the criteria of the test, regardless of a hypothetical misalignment of the machine isocenters. Granted this baseline value should be tightly established upon initialization of a program; baseline comparisons during periodic test‐to‐test review could still fail to identify a slow trend to poor congruency.

For these reasons, this retrospective isocenter congruency test with emphasis on absolute tolerance determination via relative mechanisms was developed to combine the benefits of examining both absolute and baseline results and to establish a meaningful tolerance value that reflects normal day‐to‐day functioning of a linear accelerator with a value appropriate for the demands of an SRS program. The result of this examination was a two‐tier system inclusive of a scheduled action level and an immediate action level.

In this study, we noted in our uncorrected data that two data points (i.e., dates) exceeded the action level of 1.50 mm, while 42 data points exceeded the warning level of 1.25 mm. We presented the raw data that one would expect to encounter when performing this frequent test, including setup uncertainties. The definition of the warning and action levels presented in this report for this linear accelerator are based on the approximation that 68% of our data points will fall within 1 SD and 95% of our data points will fall within 2 SDs, which requires a normal data distribution. This indicated that 1 of the 20 tests (5%) would trigger an action level. We also noticed a demonstrable offset of the isocenter on average that indicated a systematic issue that likely stemmed from the predilection to define the laser‐defined isocenter to be with the gantry, couch, and collimator in a zero degrees position. The analysis of datasets of the nature performed for this study revealed the repeated application of this conjectured bias, allowing the physicists performing this test to be more mindful of balancing this offset for all available gantry angles. This systematic error and the efficacy in the potential correction of this bias will constitute the subject of further investigation.

We determined that 71.8% of our data points fell within 1 SD (calculated based on assumption) and 98.7% of our data points fell within 2 SDs. This 5.6% and 3.9% error may be attributed to the deviation that our actual data possessed from a true Gaussian distribution. Based on the conservative estimate that our assumption provided, it was recommended to expect action levels 1 out of 20 measurements and to prepare the treatment schedule to reflect this intermittent interruption.

## V. CONCLUSIONS

To establish clear tolerance threshold values for daily implementation of the isocenter congruency test, we recommend using both the daily deviation of the test results in addition to the uncertainties in the reproducibility of setup of the test tool. That is, tolerance values should reflect daily positioning errors in setting up the test device, as well as statistical deviations in the measurements made from images comprising the datasets for each test. These tolerance values should be balanced to allow for daily setup deviations while ensuring reliable detection of true misalignments between the various isocenters in a linear accelerator treatment room.

A schedule action level and an immediate action level have been defined based on the examination and quantification of the fundamental limitations of the radiation‐defined isocenter congruency versus the various other defined isocenters for a standard linear accelerator (couch, collimator, MLC, laser‐defined, gantry). The schedule action level is 1.25 mm, while the immediate action level is 1.50 mm. These levels are established as absolute values based on machine‐specific data relative to baseline. This study proposes and demonstrates a method for assigning linear accelerator‐specific and clinically meaningful tolerance values for isocenter congruency tests that has bases in both absolute analysis for appropriate interpretation of results and relative comparison to baseline for the purpose of monitoring and responding to possible trend behaviors.

## ACKNOWLEDGMENTS

We acknowledge Norton Healthcare for their continued support, as well as the Associates in Medical Physics, LLC.

## Supporting information

Supplementary Material
