# Supplementary figures and images for: Quantifying isocenter measurements to establish clinically meaningful thresholds
Source: J Appl Clin Med Phys. 2015 Mar 8;16(2):175–88. doi: 10.1120/jacmp.v16i2.5183 (PMC5690087; doi:10.1120/jacmp.v16i2.5183)

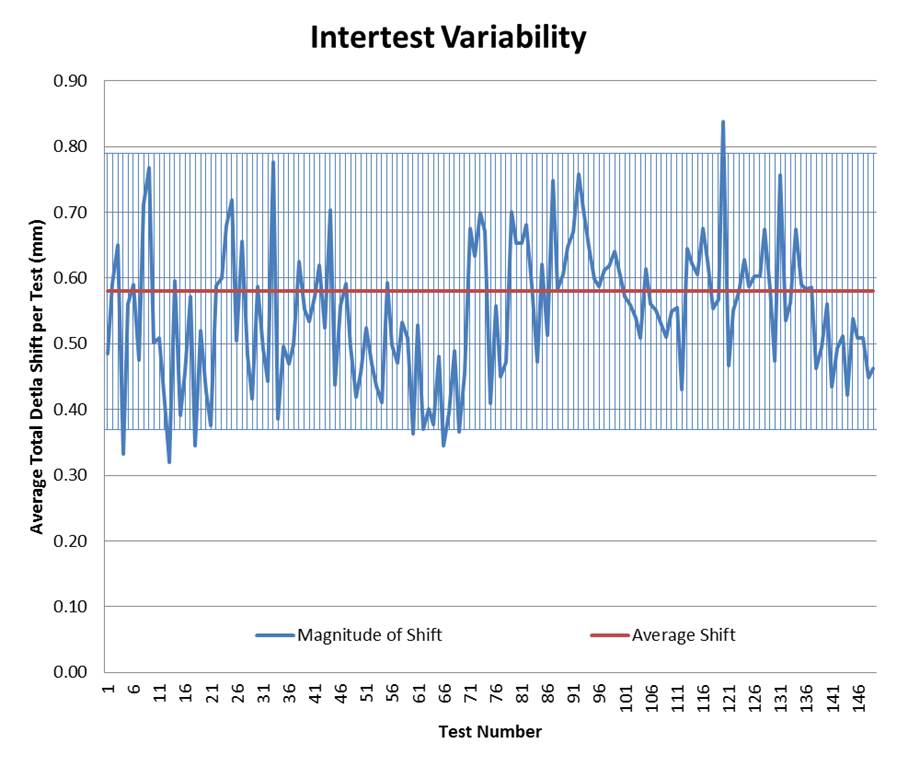

Supplement: Supplementary file 1 — Supplementary Material [file ACM2-16-175-s001.jpg]
